# Influence of Ultrasonic Activation on the Physicochemical Properties of Calcium Silicate-Based Cements

**DOI:** 10.1155/2021/6697988

**Published:** 2021-01-27

**Authors:** Fredson Márcio Acris De Carvalho, Yara Teresinha Corrêa Silva-Sousa, Carlos Eduardo Saraiva Miranda, Paulo Henrique Miller Calderon, Ana Flávia Simões Barbosa, Luciana Martins Domingues De Macedo, Fuad Jacob Abi Rached-Junior

**Affiliations:** ^1^School of Dentistry, Amazonas State University, R. Bloco Um e Três, 4-40-Platô do Piquiá, Manaus, AM 69850-000, Brazil; ^2^School of Dentistry, University of Ribeirão Preto, Av. Costábile Romano, 2201-Nova Ribeirânia, Ribeirão Preto, SP 14096-900, Brazil

## Abstract

**Purpose:**

To evaluate the influence of ultrasonic activation on the physicochemical properties of setting time (ST), flow (FL), dimensional change (DC), and solubility (SL) of the cements: MTA, MTA Repair HP, and Biodentine®.

**Materials and Methods:**

Two experimental groups were formed according to the cement activation protocol: without ultrasonic activation and with ultrasonic activation. Cements were manipulated according to the manufacturers' instructions. Ultrasonic activation group was performed with an E1 insert at power 3 (24–32 kHz) for 30 s directly in the center of the cement mass. The molds for analysis of the physicochemical properties were filled out and evaluated according to specification No. 57 from ANSI/ADA. The results were analyzed using the ANOVA test (two-way), complemented by Tukey's test (*α* = 0.05). The distilled water used during the solubility test was submitted to spectrometry to verify the release of calcium ions. The morphologies of the external surface and the cross-section of the samples were analyzed by means of a scanning electron microscope (SEM).

**Results:**

For the ST, ultrasonic activation reduced the values of MTA, MTA Repair HP, and Biodentine (*P* < 0.05). For the FL, ultrasonic activation did not alter the flow of MTA (*P* > 0.05); however, it increased the flow MTA Repair HP and Biodentine (*P* < 0.05). For the DC, the percentage values of dimensional change were higher when there was ultrasonic activation in all repair cements (*P* < 0.05). For SL, there was a reduction in the percentage of the values in MTA and MTA Repair HP (*P* < 0.05); however, there was no change in the values of Biodentine (*P* > 0.05). Ultrasonic activation favored the release of calcium ions from all cements. The SEM analysis showed, in general, that the ultrasonic activation reduced and altered the particle shape of the cement.

**Conclusions:**

The ultrasonic activation interfered in the ST, DC, FL, ultrastructural morphology, and calcium release of the repair cements. However, it did not affect the solubility of Biodentine®.

## 1. Introduction

For many years, Mineral Trioxide Aggregate (MTA) (Angelus Ind. Prod. Odontológicos, Londrina, Brazil) has been the most used repair cement in filling retrograde cavity, due to its biological properties [1, 2]. However, one of the disadvantages of this cement is the difficulty of incorporating the powder into the liquid, as well as its sandy working consistency, which negatively interferes with its insertion into the retrograde cavities or perforations [3]. To overcome these limitations, its second-generation MTA Repair HP (Angelus Ind. Prod. Odontológicos, Londrina, Brazil) was launched in the market, the main difference being that it is liquid, composed of a plasticizer used at the time of manipulation [4]. In both types of cement, manipulation is performed manually using a spatula and a glass plate, unlike Biodentine( (Septodont, Saint-Maur-des-Fossés, France), where the powder is mixed with the liquid inside a capsule, mechanically, with the aid of an amalgamator [5, 6].

The mechanical manipulation of cements can provide a uniform and homogeneous mixture between powder and liquid, and the reduction of the particles' size [7, 8] provides greater interaction of the cement with dentin [9], increasing the contact area between them during the hydration process [10]. These processes can also allow greater interaction with dentin and, consequently, a deeper penetration into the dentinal tubules and material adaptation to the retrograde cavity [9]. When it comes to MTA cement, the literature has shown some controversial results. Its mechanical manipulation leads to the increase of the microhardness [10], interfering in the solubility [11]. On the other hand, the mechanical manipulation, apparently, does not influence the physical and chemical properties of setting time, pH, flow, dimensional change, and film thickness [11, 12].

Ultrasonic agitation is based on the transmission of acoustic energy from an insert to a liquid by ultrasonic waves [13]. This process promotes the swirling of the irrigating solution and, concomitantly, increases and decreases the hydrostatic pressure, promoting the generation of cavitation bubbles that implode, making temperature solution augment [13]. This phenomenon provides a greater ability to remove the smear layer and debris from the root canal interior [13]. In this way, it probably favors deeper penetration of cement inside the dentinal tubules, improving the material union resistance to the root dentin [14].

Thus, given the development of a repair material that brings a perspective of improvement in its handling and consistency, as well as the possibility of different handling techniques, this study aimed to evaluate the influence of ultrasonic agitation on the physicochemical of repair cement: MTA, MTA Repair HP, and Biodentine(. The null hypothesis in this study was that ultrasonic activation would interfere with the physicochemical properties of repair cement.

## 2. Materials and Methods

In this study, three types of repair cements were used: MTA (Angelus Ind. Prod. Odontológicos, Londrina, Brazil), MTA Repair HP (Angelus Ind. Prod. Odontol., Londrina, Brazil), and Biodentine( (Septodont, Saint-Maur-des-Fossés, France). The cements were manipulated according to two techniques: without ultrasonic activation and with ultrasonic activation. The physicochemical properties of setting time, flow, dimensional change, and solubility were performed according to ANSI/ADA standards. Two experimental groups were formed according to the cement activation protocol.

Group I, without activation: the cement was handled according to the manufacturer's instructions and the molds for each physicochemical property to be evaluated were filled.

The MTA was handled with the powder portion (0.14 g) added to a drop of distilled water, dispensed through a dosing bottle positioned vertically to the glass plate, and spatulated with a 24F spatula (Duflex, Rio de Janeiro, RJ, Brazil) for 40 s to obtain a homogeneous mixture.

The MTA Repair HP was made with a 0.085 g portion of the powder, corresponding to a capsule, dispensed on a glass plate, and a drop of its liquid was added, obtained through the dosing bottle positioned vertically to the glass plate. The powder was agglutinated and manipulated into the liquid for 40 s, using a 24F spatula until a homogeneous mass was obtained.

For the manipulation of Biodentine(, 5 drops of its liquid were dispensed in the capsule containing cement powder and this was placed in an Astronmix amalgamator (Dabi Atlante, Ribeirão Preto, SP, Brazil) and 2 cycles of 15 s were performed with a frequency of 60 Hz.

Group II, with activation: all cements were manipulated as described in Group I; then, they were activated using an ultrasound applied directly in the center of the mixture for 30 s. A noncutting insert (# 20, taper 0.01) (E1-Irrisonic, Helse Dental Technology, Santa Rosa de Viterbo, SP, Brazil) coupled with the piezoelectric ultrasonic unit (P100, EMS-Electro Medical System, Switzerland) at 30% power, with frequency at 24–32 Hz [8–10], was used. The MTA was put in a sterile plastic capsule, whereas for the MTA Repair HP and Biodentine, their respective capsules were used. It is worth noting that during the activation of the cement mass, contact between the insert and the capsule was avoided.

### 2.1. Setting Time

Five circular Teflon molds (Polytetrafluoroethylene; DuPont, HABIA, Knivsta, Sweden) with an internal diameter of 10 mm and a thickness of 2 mm were prepared for each group. The molds were fixed with wax on a glass plate, filled with cement soon after preparation, and transferred to an incubator with 95% relative humidity and 37°C. After 120 ± 10 s from the onset of mixing, a Gilmore-type needle with 100 ± 0.5 g and a flat end of 2.0 ± 0.1 mm in diameter was carefully lowered vertically onto the horizontal surface of the testing sample. The placement of the Gilmore needle on the material was repeated at 60 s intervals, until it no longer caused marks on the cement tested. The time used from the start of mixing to this point was recorded. The setting time of each group was determined by calculating the arithmetic mean of the five repetitions. If the results differed by more than 5%, the test was repeated. It is worth mentioning that regardless of the ultrasonic activation, the setting time counting was started 120 ± 10 s after the mixing procedure ending.

### 2.2. Flow Test

A total of 0.5 mL of cement was placed on a glass plate using a graduated disposable 3 mL syringe. At 180 ± 5s after the onset of mixing, another glass plate with a mass of 20 ± 2 g and a load of 100 g was applied centrally on top of the material. Ten minutes after the mixing started, the load was removed and the average of the major and minor diameters of the compressed discs was measured using a digital caliper (Mitutoyo MTI Corporation, Tokyo, Japan). For this test, five replicates of each experimental group were performed.

### 2.3. Dimensional Change

Five cylindrical Teflon molds (3.57 mm height × 3.0 mm in diameter) were placed on a glass plate covered with cellophane and fixed with utility wax. The molds were filled with the cement being studied and then a microscope slide, also covered with cellophane, was placed onto the molds, making a slight digital pressure. Five minutes after the beginning of the mixture, the cement was taken to the incubator at 37°C and 95% relative humidity for a period corresponding to three times the setting time of each tested cement. The samples were removed from the mold and their lengths were measured with the aid of a digital caliper, thus obtaining their initial length. The cement samples were then placed in plastic containers containing 2.24 mL of distilled and deionized water and taken to the incubator at 37°C and 95% humidity for 30 days. After this period, the samples were removed from the containers and dried with absorbent paper, and their lengths were measured again using a digital caliper, thus obtaining the final length of the samples. The percentage of dimensional alterations was calculated by using the following formula: L30-L/*L* × 100, where L30 is the length of the sample after 30 days of storage and *L* is the initial length of the sample.

### 2.4. Solubility Test

Ten circular Teflon molds were made with 1.5 mm thickness and 7.75 mm internal diameter, measures recommended by Carvalho-Júnior et al. [15] based on ANSI/ADA Specification No. 57. Each mold was positioned on a glass plate wrapped in cellophane film and filled with the cement to be tested. A nylon thread was included in the cement mass and another glass slide, also wrapped in cellophane, was positioned over the mold. The assembly was pressed manually until the plates touched the surface of the mold evenly. The sets formed by the Teflon molds, glass plates, nylon threads, and cement were transferred to an incubator at a temperature of 37°C and 95% relative humidity, for a time interval three times greater than the setting time of each cement. The samples were then removed from the molds and weighed two by two on an HM-200 precision scale (A&*D* Engineering, Inc., Bradford, MA, USA) adjusted to 0.0001 g, to obtain the initial weight. The samples were suspended by nylon threads and placed two by two in cylindrical plastic containers, containing 7.5 mL of distilled and deionized water, taking care to avoid the contact between the samples and the inner surface of the container. The containers with the samples were taken to the incubator at a temperature of 37°C and 95% relative humidity for seven days. After this period, the samples were removed from the liquid, rinsed with distilled and deionized water, and placed in a dehumidifier containing 98% H_2_SO_4_ for 24°h. Then, the samples were weighed again two by two to obtain the final weight. The weight loss of each sample (initial mass minus final mass) was expressed as a percentage of the original mass (m% = mi–mf) and taken as the solubility of the sealer [16–19].

### 2.5. Statistical Analysis

For each test, five replicates of each experimental group were performed, and the means were statistically compared. The Kolmogorov–Smirnov test showed that the results were consistent with a normal distribution curve. The parametric statistical analysis was performed (two-way analysis of variance [ANOVA] and post hoc Tukey's test), and the significance level was set as 5% (GraphPad InStat; GraphPad Software, Inc., San Diego, CA).

### 2.6. Flame Atomic Absorption Spectrometry (FAAS): Quantification of Released Ca^2+^ Ions

The immersion liquids of the specimens were analyzed by FAAS (Varian, Palo Alto, CA, USA) to quantify the Ca^2+^ ions. To prepare calcium standard solutions, a stock solution (Merk, Darmstadt, Germany) with a concentration of 1000 *μ*g mL^−1^ was used. The metal analytical curve was obtained from the appropriate dilutions of the stock solution. The concentration range of the calcium standard solutions was 0.5, 1.0, 2.0, 3.0, 4.0 and 5.0 mg/L. The immersion liquids of the specimens were diluted before the analysis, depending on the metal concentration level in the sample.

### 2.7. Scanning Electron Microscopy

Ten circular samples of 1.5 mm × 7.75 mm internal diameter of each studied cement type were prepared as described for the solubility test. Scanning electron microscopy analysis was performed on both submitted (*n* = 5) and nonsubmitted (*n* = 5) to the solubility test. The samples of each group were sectioned into halves with a disposable surgical scalpel blade. Afterward, the samples were fixed with double-sided adhesive tape (3M, São Paulo, SP, Brazil) on a stub and taken to the metallizer (Bal-tec AG, Balzers, Germany) to be covered by a thin layer of the gold-palladium alloy. The analysis was performed using a scanning electron microscope JSM-6610 LV (JEOL Ltd, Tokyo, Japan) with 25 kV. The external and internal surfaces of the samples were analyzed before and after immersion step at 500× magnification in backscatter mode [16].

## 3. Results

In Table 1, the results of the analysis of the physicochemical properties and the release of Ca^2+^ ions from repair cements according to the different agitation protocols are presented.

### 3.1. Setting Time

Ultrasonic activation reduced the setting time of MTA, MTA Repair HP, and Biodentine cement (*P* < 0.05).

### 3.2. Flow

Ultrasonic activation did not alter the flow of MTA (*P* > 0.05); however, it increased both MTA Repair HP and Biodentine® flow (*P* < 0.05).

### 3.3. Dimensional Change

The percentage values of dimensional change were higher when there was ultrasonic activation in all repair cements (*P* < 0.05).

### 3.4. Solubility

In the group with ultrasonic activation, there was a reduction in the percentage of the values of MTA and MTA Repair HP (*P* < 0.05); however, there was no change in the values of Biodentine® (*P* > 0.05).

### 3.5. Flame Atomic Absorption Spectrometry (FAAS): Quantification of Released Ca^2+^ Ions

The analysis by atomic absorption spectrometry of the liquids showed that the Biodentine® had a significant release of Ca^2+^ ions, higher than other cements, regardless of the use of ultrasound.

### 3.6. Scanning Electron Microscopy

**Figure 1:**
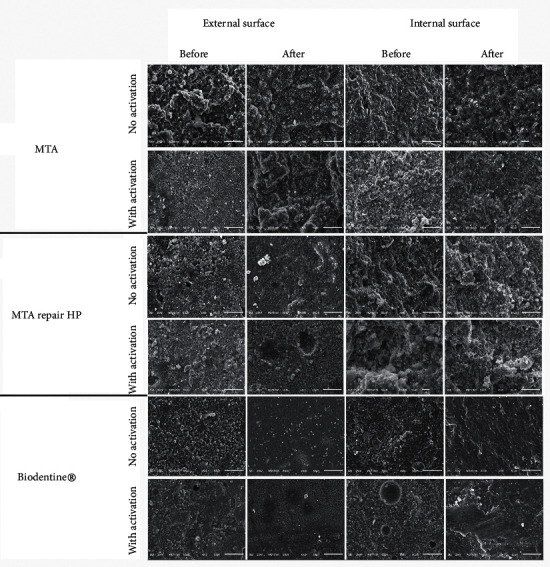
Electromicrographs (500 × magnification) of the external and internal surfaces of repair cements with or not ultrasonic activation, before and after solubility test.

Surfaces with particles of heterogeneous size and shape and the presence of crystalline and amorphous phases (Figure 1) were distinguished before immersion in water on the electromicrographs of MTA and MTA Repair HP samples without ultrasonic activation. After the immersion, the structure of the external and internal surfaces was more compact, with increased deposition of the amorphous phase. When ultrasonic activation was performed on the external and internal surfaces, there was a reduction in the size of particles and greater proximity between them, both before and after immersion (Figure 1). Biodentine®, in general, presented only the amorphous phase. In the group without ultrasonic activation (Figure 1), on the external surface before immersion, regions with compact appearance and other areas composed of particles of different sizes and shapes were evidenced. On the internal surface, a dense structure was observed, with rounded areas circumscribed by whitish lines, suggestive of bubble formation. After immersion, little ultrastructural variation was noted. When ultrasonic activation was performed (Figure 1), little variation in the size and shape of its particles was perceived. However, on the entire external surface, after immersion, the presence of a stratified network formed by interconnected crystals was verified, referring to a more cohesive and uniform structure. In its internal surface, after immersion, a more dense and homogeneous structure was discerned.

## 4. Discussion

The setting time was the first test to be performed, as it depends on the chemical composition, the particle size of the materials, the ambient temperature, and the relative humidity of the air [20]. This is considered the most important physicochemical property since the other properties depend on this variable [17]. The ANSI/ADA [18] established that endodontic cement should not have a setting time greater than 10% of that determined by the manufacturer. Concerning the protocol without activation, the Biodentine® setting time was shorter than that of MTA, probably due to the presence, in its formulation, of calcium chloride, which, being soluble in water, accelerates its prey [19]. In the case of MTA Repair HP, it is assumed that the plasticizer inserted in the liquid formulation of this cement favored the reduction of the setting time. Thus, the analysis of the results obtained in the present study allowed us to conclude that the MTA and Biodentine® were in accordance with the standards when handled according to the manufacturers' guidelines. However, when activated by ultrasound, the setting time was reduced, which was outside the ANSI/ADA specification.

When using ultrasonic activation, which is based on the transmission of acoustic energy from an insert to the material using ultrasonic waves [13], a whirlwind occurs and, at the same time, an increase and reduction in hydrostatic pressure, which promotes the formation of cavitation bubbles that implode and produce an increase in the temperature of the material [13]. The increase in temperature in the endodontic cement mass interferes with the physicochemical properties, as it increases the reaction kinetics [21]. Additionally, ultrasonic waves, when in contact with the material, cause the dispersion of the particles, leading to an increase in the contact surface between them, providing a more efficient homogenization and, therefore, greater interaction and accommodation of these particles, in addition to favoring a greater diffusion of water in the environment, which provides a higher degree of hydration and, consequently, a higher reaction speed [7, 10].

In this sense, the importance of hydration in the setting process of MTA and MTA Repair HP is observed, occurring through an exothermic reaction, which requires the hydration of the cement powder to obtain the final product. Thus, the MTA's prey depends on water, whose presence leads to the conversion of calcium oxide into calcium hydroxide [22]. The hydration process of calcium silicate-based cements is a highly complex phenomenon that can significantly influence the biological, chemical, and physical properties of the final product [23]; therefore, greater availability of water in the environment would favor the reactions of these cements, reducing the setting time. The same occurs with the setting of Biodentine®, which occurs immediately after mixing the powder with the liquid by grinding, so that the calcium silicate particles react with the water of a solution with a high pH, containing Ca^2+^, OH^−^, and SiO_4_^4-^ ions [6, 24]. During the reaction, the hydrated calcium silicate gel formed in the chemical reaction undergoes polymerization and forms a solid structure, while an increase in the alkalinity of the medium occurs due to the release of calcium hydroxide [25]. The actions of calcium chloride and water-soluble polycarboxylate, as a prey accelerator and water reducing agent, respectively, promote the acceleration of hydration and crystallization and, therefore, allow quicker setting time [19, 22].

For the flow test, ANSI/ADA determines that the cement must not have a diameter of less than 20 mm. In this study, regardless of the activation protocol, no cement met the standard. However, when ultrasonic activation was used, there was an increase in the average value of the diameter of the repaired cements. Furthermore, according to Duque et al. [11], the reduction of the cement particle size increases the contact surface available for the reaction with water during the hydration process, which was also noticed in the present study by using SEM analysis. Thus, the change in the hydration process during the chemical reactions of calcium silicate-based cements can interfere with the flow of these cements [23]. Additionally, the increase in temperature [21], associated with the reduction of the particle size, could contribute to the reduction of the viscosity of the cements, favoring the flow.

The dimensional change has a great influence on the stability of cements since when undergoing contraction, the sealing capacity of endodontic cements is reduced, enabling the installation of infectious processes [26]. On the other hand, the expansion of these cements could cause adhesive failures between the cement and dentin [26]. Thus, ANSI/ADA established that, during the dimensional change test, the contraction of the materials should not exceed 1% and the expansion should not exceed 0.1%. The results obtained in this study indicated that the tested cements were not in accordance with ANSI/ADA standards, regardless of ultrasonic activation. According to Shahi et al. [12], the dimensional change is directly related to the material's setting time, so that its decrease causes an increase in the dimensional change values. In this study, this fact occurred when the cements were subjected to ultrasonic activation so that there was a decrease in the setting time that may have favored the expansion of the cement specimens. It is also believed that the movement of the particles and the reduction in the size of the particles, as verified in the analysis by SEM, provided greater expansion, probably due to the absorption of water that occurred in the hydration process of the cements.

The greater the powder surface area, the greater the tendency of the cement particles to react with water, incrementing the hydration process and, as a consequence, the setting time was reduced. In a report by Saghiri et al. (2020), the sol-gel method resulted in finer powder with a narrower range of particles and uniform powder particle distribution when compared with mechanical activation promoted by milling. Despite this fact, a particle size reduction was clearly observed when the second method was used. These authors also selected SEM to evaluate the size of particles and its reduction was verified, which, additionally, allowed a slight decrease in the setting time of the studied cements.

For the solubility test, ANSI/ADA states that the solubility should not exceed 3% of the mass of the material. In the group without ultrasound activation, Biodentine® showed a loss of mass, whereas MTA and MTA Repair HP had an increase in mass due to water absorption. Therefore, only Biodentine® is within the standards required by ANSI/ADA, regardless of ultrasonic activation, which corroborates the results obtained by Grech et al. [19], who also found a decrease in mass. It is believed that since this cement contains tricalcium silicate, during its industrial process, particles with greater uniformity are obtained [27]. In addition, its manipulation is mechanically carried out through crushing, which already provides an approximation of the cement particles and, possibly, it allows the formation of a more compact structure, as shown in the analysis by SEM. Therefore, this structure is more resistant to solvent attack, reducing the loss of material [28]. The increase in mass obtained by MTA and MTA Repair HP can be explained by the cement hydration process. During the hydration process, there is an increase in mass, which forms pores on the surface and inside the product, being filled by the water left over inside the product matrix [26]. In this way, these empty spaces make the cement structure weaker, susceptible to the action of the solvent, making water absorption easier [29]. This reinforces the idea that ultrasonic activation has provided a reduction in the cement particle dimensions and, at the same time, greater proximity among them. Thus, it is believed that it also favored the reduction of the pore size and, consequently, the reduction of water absorption.

In the analysis by spectrometry of the immersion liquids, all cements tested released calcium ions. This fact can be explained by the dissociation of calcium hydroxide, a byproduct originated from the hydration of tricalcium or dicalcium silicate, in Ca^2+^ and OH^−^ ions [30]. Moreover, in Biodentine®, there is calcium chloride in its chemical composition, which favors the release of calcium ions [28]. The ultrasonic activation protocol promoted an increase in the release of calcium, possibly due to the movement of the particles that favored the dispersion of the particles in the cement mass. It is believed that, in Biodentine®, greater release occurred due to the presence of calcium chloride associated with the movement of particles promoted by ultrasonic activation.

It was demonstrated that the ultrasonic activation interfered with the physicochemical properties and the ultrastructural morphology of the repair cements. Despite these results, the evaluation of the advantages and disadvantages of using ultrasonic activation considering each repair cement should be encouraged.

## 5. Conclusions

The ultrasonic activation interfered in the setting time, dimensional change, flow, ultrastructural morphology, and calcium release of the repair cements. However, it did not affect the solubility of Biodentine®.

## Figures and Tables

**Table 1:** Physicochemical properties and Ca^2+^ ion release of repair cements without activation and with ultrasonic activation protocol (mean ± standard deviation).

Repair cements	Protocol activation	Setting time (min)	Flow (mm)	Dimensional change (%)	Solubility (%)	Ca^2+^ ions release (mg/L)
MTA	No activation	14.20 ± 0.83A	14.39 ± 0.22A	0.71 ± 0.14A	−15.53 ± 1.98A	27.96 ± 1.54
	Ultrasonic	11.00 ± 0.70B	15.74 ± 0.44A	5.22 ± 0.85B	−5.30 ± 0.59B	33.35 ± 2.80

MTA Repair HP	No activation	12.20 ± 1.09A	9.98 ± 0.18A	1.72 ± 0.62A	−3.66 ± 1.01A	28.76 ± 0.93
	Ultrasonic	10.00 ± 0.70B	10.95 ± 0.14B	3.67 ± 1.97B	−0.86 ± 0.89B	35.85 ± 5.07

Biodentine(	No activation	12.80 ± 1.30A	9.84 ± 0.13A	0.61 ± 0.14A	0.31 ± 0.22A	225.43 ± 69.64
	Ultrasonic	10.40 ± 0.89B	10.59 ± 0.18B	2.30 ± 0.69B	0.43 ± 0.12A	1.105.15 ± 298.24

^*∗*^Distinct letters in the same column indicate statistically significant difference between groups (Tukey, *p* < 0.05).

## Data Availability

No data were used to support this study.
